# *Nr2f2* Overexpression Aggravates Ferroptosis and Mitochondrial Dysfunction by Regulating the PGC-1*α* Signaling in Diabetes-Induced Heart Failure Mice

**DOI:** 10.1155/2022/8373389

**Published:** 2022-08-30

**Authors:** Weilun Miao, Mengli Chen, Minglong Chen, Chang Cui, Yue Zhu, Xinping Luo, Bangwei Wu

**Affiliations:** ^1^Department of Cardiology, Huashan Hospital Affiliated to Fudan University, 200000, No. 12, Urumqi Middle Road, Shanghai, China; ^2^Department of Cardiology, The First Affiliated Hospital of Nanjing Medical University, 210000, No. 300, Guangzhou Road, Nanjing, Jiangsu, China

## Abstract

Diabetes is well recognized to increase the risk of heart failure, which is associated with higher mortality and morbidity. It is important for the development of novel therapeutic methods targeting heart failure in diabetic patients. Ferroptosis, an iron-dependent regulated cell death, has been implicated in the progression of diabetes-induced heart failure (DIHF). This study was designed to investigate the contribution of Nr2f2 to the activation of ferroptosis and mitochondrial dysfunction in DIHF. We established a diabetic model by a high-fat feeding diet combined with an intraperitoneal injection of streptozotocin. After 16 weeks, Nr2f2 expression was increased in heart tissue of DIHF mice. *In vivo*, DIHF mice overexpressing Nr2f2 (AAV9-cTNT-Nr2f2) exhibited severe heart failure and enhanced cardiac ferroptosis compared with DIHF control mice (AAV9-cTNT-ctrl), accompanied by mitochondrial dysfunction and aggravated oxidative stress reaction. *In vitro*, Nr2f2 knockdown ameliorated ferroptosis and mitochondrial dysfunction by negatively regulating PGC-1*α*, a crucial metabolic regulator. PGC-1*α* knockdown counteracted the protective effect of Nr2f2 knockdown. These data suggest that Nr2f2 promotes heart failure and ferroptosis in DIHF by modulating the PGC-1*α* signaling. Our study provides a new idea for the treatment of diabetes-induced heart failure.

## 1. Introduction

Heart failure (HF) is a major cause of cardiovascular morbidity and mortality in individuals with diabetes, including type 1 diabetes (T1DM) and type 2 diabetes (T2DM), which is the main clinical manifestation of diabetic cardiomyopathy (DCM) in end-stage describing myocardial dysfunction in the absence of hypertension, atherosclerotic heart disease, and valvular heart disease in patients with diabetes. Globally, an estimated 700 million people are expected to be living with diabetes by 2045. The understanding of pathophysiology and underlying mechanism of diabetes-induced heart failure (DIHF) is still limited. Due to the rapidly increasing prevalence of diabetes worldwide, there is a need to better understand the pathogenesis of DIHF and develop novel therapies [1].

Evidence suggests that metabolic disturbance, inflammation, oxidative stress-induced cardiomyocyte death (including apoptosis, autophagy, pyroptosis, ferroptosis, and necrosis), and mitochondrial dysfunction have a crucial role in cardiac remodeling in DIHF [2, 3]. Ferroptosis is an iron-dependent cell death, characterized by iron overload and lipid peroxidation. It has been proved that ferroptosis may be involved in the pathogenic process of several cardiovascular diseases, including myocardial ischemia-reperfusion injury [4–7], doxorubicin-induced cardiomyopathy [8], septic cardiomyopathy, heart failure [9], inflammatory responses after cardiac transplantation [10], and vascular injury [11]. Recent studies have shown that ferroptosis is essential for DCM [12]; however, the underlying mechanism remains to be elucidated.

Nuclear receptor subfamily 2, group F, member 2 (Nr2f2) (also known as COUP-TFII), belongs to a family of nuclear orphan receptors. Nr2f2 plays a significant role in energy metabolism, stem cell differentiation, tumor progression, and the development of the cardiovascular system [13]. Nr2f2 is expressed at low levels in the adult heart [13], but expression increased under certain pathological conditions, such as heart failure [14]. In diabetes, Nr2f2 may be involved in the regulation of glycometabolism, oxidative stress, and inflammation in the pancreas [15]. Previous studies have demonstrated that elevated Nr2f2 expression is generally accompanied by mitochondrial dysfunction, increased reactive oxygen species (ROS) production, and alterations in energy metabolism [13, 16, 17]. Although the role of mitochondria in ferroptosis has been controversial, the occurrence of ferroptosis is always accompanied by abnormal mitochondrial dysfunction. Evidence suggested that inhibition of mitochondrial TCA cycle or electron transfer chain mitigated mitochondrial membrane potential hyperpolarization, lipid-ROS accumulation, and ferroptosis [18]. Hence, we hypothesized that Nr2f2 might participate in the regulation of ferroptosis in DIHF.

In this study, we investigated the contribution of Nr2f2 to the cardiac function of diabetic mice and the activation of ferroptosis *in vivo* and *in vitro*. Findings showed that Nr2f2 expression was increased in heart tissue of diabetic mice by feeding a high-fat diet and streptozotocin intraperitoneal injection. *In vivo*, overexpressing Nr2f2 exhibited symptoms of severe heart failure, ferroptosis, mitochondrial dysfunction, and oxidative stress in diabetes mice. *In vitro*, Nr2f2 knockdown ameliorated ferroptosis and mitochondrial dysfunction in palmitic acid- (PA-) treated neonatal rat cardiomyocytes (NRCMs). Mechanically, Nr2f2 promoted ferroptosis and mitochondrial dysfunction by regulating PGC-1*α* negatively. These data suggest that Nr2f2 is associated with diabetes-induced heart failure and ferroptosis by modulating the PGC-1*α* signaling pathway, providing a novel therapeutic target.

## 2. Materials and Methods

### 2.1. Animal Model

All animal procedures were approved by the animal care and use committee of Huashan Hospital of Fudan University (no. 201802049S). Male C57BL/6 mice (weight approx. 20 g, age 4–5 weeks) were obtained from the animal experimental center of Fudan University. Mice were housed in a specific pathogen-free animal facility with a standard temperature range and feeding regimen. Prior to feeding a high-fat (D12492, 60% kcal fat, Research Diets Company, NJ, USA) or normal diet, fasting blood glucose levels (Roche, Mannheim, Germany) and body weight were measured. Con (control) mice and DIHF mice were fed a normal or high-fat diet for one month; subsequently, Con and DIHF mice received an intraperitoneal injection of diluent or 100 mg/kg streptozotocin (STZ) (Sigma-Aldrich, San Luis, MO, USA). Fasting blood-glucose levels were measured to confirm diabetes (fasting blood glucose > 11 nmol/L). DIHF mice were fed a high-fat diet for a total of 4 months, and the fasting blood-glucose level and body weight were measured once a month.

### 2.2. Viral Construction and Delivery

The biological function of Nr2f2 was determined by cardiac-specific overexpression of Nr2f2 in mice by transfection of adeno-associated virus 9 (AAV-9). Purified AAV-9 encoding Nr2f2 was generated by Hanheng Biotechnology (Shanghai, China). Mice were divided into four groups: AAV9-cTNT-ctrl-Con, AAV9-cTNT-ctrl-DIHF, AAV9-cTNT-Nr2f2-Con, and AAV9-cTNT-Nr2f2-DIHF. 8 weeks after the STZ injection, DIHF mice received an intravenous tail injection of 100 *μ*L 5.0–6.5 × 10^13^ GC/mL AAV9-cTNT-Nr2f2 and Con mice received an intravenous tail injection of 100 *μ*L 5.0–6.5 × 10^13^ GC/mL AAV9-cTNT-ctrl.

### 2.3. Echocardiography

After 4 months of a high-fat/normal diet, cardiac functioning was evaluated using echocardiography (Vevo 2100; Visual Sonics, Toronto, Canada). Mice were anesthetized with 2% isoflurane. Ejection fraction (EF%), fractional shortening (FS%), left ventricular diastolic diameter (LVDd, mm), and left ventricular systolic diameter (LVDs, mm) were measured.

### 2.4. Histology

Heart tissue was fixed with 4% PFA, embedded in paraffin, and cut into 5 mm sections for hematoxylin & eosin (H&E) staining. H&E staining was performed according to a protocol of the H&E staining kit (Solarbio, G1120, China). The heart sections were also subjected to Masson staining using a Masson Trichrome staining kit (Solarbio, G1346, China) according to the manufacturers' instructions. 4-4-HNE immunohistochemistry staining was conducted to measure the lipid peroxidation product 4-hydroxynonenal (1 : 200, ab46545, Abcam, Hong Kong, China). Images were captured using a Nikon Eclipse microscope (Nikon, Tokyo, Japan) and NIS Elements software.

### 2.5. Transmission Electron Microscope

The morphology of mitochondria was observed using a transmission electron microscope (TEM). Heart tissue was fixed with 2.5% glutaraldehyde for 1 hour at room temperature and then overnight at 4°C. Fixed tissues were embedded in Embed-812 resin and sectioned into 1 mm^3^ blocks. Sections were stained with uranyl acetate and lead citrate for 30 and 15 min, respectively. Ultrathin sections were observed using a JEOL JEM-2100 transmission electron microscope (Tokyo, Japan).

### 2.6. Isolation of Neonatal Rat Ventricular Cardiomyocytes (NRCMs)

NRCMs were isolated as previously described [19]. Briefly, rat hearts were dissected and minced in 1x ADS-buffered saline solution. Tissues were digested in 1x ADS-buffered saline solution containing 1.5 mg/mL collagenase type II (Worthington, Lakewood, NJ, USA). Fibroblasts and cardiomyocytes were layered using a Percoll density gradient (GE Healthcare, Uppsala, Sweden,17-0891-02). Cardiomyocytes were collected, centrifuged, and resuspended in high-glucose DMEM (Gibco, Pasadena, CA, USA) supplemented with 10% FBS (Gibco, USA) and 5% horse serum (HyClone, Logan, UT, USA).

### 2.7. Downregulation and Upregulation of Target Genes

Nr2f2 overexpression plasmid was constructed by Jikai Genochemical Technology (Shanghai, China). Nr2f2 small interfering RNA (Nr2f2-siRNA) and PGC-1*α* small interfering RNA (PGC-1*α*-siRNA) were designed and constructed by Jima Gene (Suzhou, China). Nr2f2-siRNA target sequences were as follows: 5′ to 3′: sense-GUGGAAAGUUUGCAGGAAATT antisense-UUUCCUGCAAACUUUCCACTT; PGC-1*α*-siRNA target sequences were as follows: 5′ to 3′: sense-GCUCUUGAGAAUGGAUAUATT antisense-UAUAUCCAUUCUCAAGAGCTT. Overexpression plasmid and small interfering RNA were transfected into NRCMs using lipofectamine 3000 (#2067450, Invitrogen, MA, USA) for 12 hours according to the manufacturer's instruction.

### 2.8. Cell Treatment

After transfection, NRCMs were exposed to serum-free DMEM containing 500 nM palmitic acid (PA) (Sigma-Aldrich, St. Louis, MO, CAS.57-10-3) for 24 hours and harvested. 2% BSA was received as control.

### 2.9. Western Blot

Heart tissue or NRCMs were lysed using RIPA buffer (89900, Thermo Fisher Scientific, Waltham, USA). Protein concentration was determined using a Pierce™ BCA Protein Assay Kit (23225, Thermo Fisher Scientific). SDS-PAGE was performed with 10% gradient gel (PG112, Epizyme, China). Proteins were transferred to a PVDF membrane (#0301004001, Roche, USA). Membrane was incubated with primary antibodies at 4°C overnight after being blocked with 2% BSA for 2 hours. Primary antibodies were as follows: Nr2f2 (#6434, 1 : 1000, CST, Boston, MA, USA, Rabbit mAb), GPX4 (ab125066, 1 : 1000, Abcam, rabbit), PTGS2 (#12282, 1 : 1000, CST, Rabbit mAb), *β*-actin (cat no. 66009-1-Ig, 1 : 5000, Proteintech, Rosemont, IL, USA, monoclonal antibody), PGC-1*α* (cat no. 66369-1-Ig, 1 : 1000, Proteintech, monoclonal antibody), and G*Α*PDH (cat no. 60004-1-Ig, 1 : 5000, Proteintech, monoclonal antibody). Secondary antibodies were as follows: anti-rabbit IgG, HRP-linked antibody (7074P2, 1 : 5000, CST), and anti-mouse IgG, HRP-linked antibody (7076, 1 : 5000, CST). Chemiluminescent reagents (17295, Thermo Fisher Scientific) and a Molecular Imager ChemiDoc™ XRS+ Imaging System (Bio-Rad, California, USA) were used for detection. Relative protein expression was quantified by ImageJ software (ImageJ 1.8.0, Bethesda, MD, USA).

### 2.10. Quantitative Real-Time PCR

RNA was extracted from heart tissue and cells as previously described. Reverse transcription was performed using a PrimeScript™ RT reagent kit (RR037A, TaKaRa Bio, Beijing, China), according to the manufacturer's instructions. cDNA was analyzed using an Applied Biosystems 7900HT Fast Real-Time PCR System (ABI) with SYBR Green Master Mix (170-8882AP, Bio-Rad), according to the manufacturers' instructions. Primer sequences of investigated genes are listed in Table 1.

### 2.11. Malonaldehyde (MDA), GSH, SOD, and Iron Content

The molecular mechanism of ferroptosis was investigated by measuring MDA, GSH content, SOD activity, and iron content. Heart tissue or NRCMs were homogenized and lysed. MDA, GSH content, and SOD activity were measured using the MDA detection kit (Beyotime, Shanghai, China, S0131S), GSH assay kit (Beyotime, Shanghai, China, S0052), and SOD assay kit (Beyotime, Shanghai, China, S0109), according to the manufacturer's instructions. The iron content was determined using an iron assay kit (ab83366, Abcam), according to the manufacturer's instructions. Data are presented as nmol/mg protein or nmol/mg wet tissue.

### 2.12. Cell Viability Assay

Cell viability was assessed using the Cell Counting Kit-8 (CCK-8) (Dojindo, Kumamoto, Japan). 200000/mL NRCMs were seeded in 96-well plates. After the indicated treatment, 10 *μ*L CCK-8 solution was added to each well and incubated for 2–3 hours. Optical density (OD 450 nm) was detected using a microplate reader (BioTek Instruments, Colmar, France). The percentage of viability was calculated by the ratio of experimental cells to control cells.

### 2.13. Cellular Lipid ROS Assay

C11-BODIPY 581/591, an indicator of lipid peroxidation, was used to detect the cellular content of lipid ROS, which is involved in ferroptosis. 100000 NRCMs/well were seeded into a 12-well plate and incubated with C11-BODIPY 581/591 (D3861, Thermo Fisher Scientific) for 20 min at 37°C. Cells were digested with 0.25% EDTA trypsin and resuspended in 1x HBBS. Lipid ROS were detected using a CytoFLEX flow cytometer (Beckman Coulter, Brea, CA, USA) at excitation wavelengths of 488 nm and 565 nm, and the data were analyzed by FlowJo software (FlowJo, Ashland, OR, USA). The oxidated C11-BODIPY (ox-C11-BODIPY FITC) fluorophore shifts the fluorescence emission from red to green. ox-C11-BODIPY FITC (%) represents the lipid-ROS percentage.

### 2.14. Mitochondrial Membrane Potential (MMP) Measurement

The mitochondrial membrane potential (∆Ψm) in NRCMs was measured using JC-1 staining (C2006, Beyotime, China). Cell samples were stained with JC-1 dye at 37°C for 20 min and washed with buffer solution 3 times. Fluorescence intensity was measured at 555 nm and 488 nm using a Zeiss inverted fluorescence microscope (Carl Zeiss, Thornwood, NY). Results were analyzed using ImageJ software (ImageJ 1.8.0, Bethesda, MD, USA) and calculated as the ratio of red to green fluorescence. A reduction in the red to green fluorescence ratio indicated mitochondrial depolarization.

### 2.15. ATP Detection Assay

The ATP content in the NRCMs was detected using the ATP assay kit (Beyotime, S0026, China), according to the manufacturer's instructions. Briefly, cell samples (10^6^ cells per 100 100 *μ*L lysis buffer) were lysed and centrifuged at 12000 × g for 5 min at 4°C to collect supernatants. Take an appropriate amount of ATP detection reagent, and dilute the ATP detection reagent with ATP detection reagent diluent in a ratio of 1 : 9. Values were measured using a luminometer (BioTek instruments, Colmar, France). The ATP concentration was calculated according to an ATP-standard curve and normalized to protein concentration of the supernatant.

### 2.16. Statistical Analysis

Statistical analysis was performed with GraphPad Prism version 8.0.2 software (GraphPad Software, San Diego, CA). Data are presented as means ± SDs. Normality of the data was explored with Shapiro-Wilk's test. For the two independent samples, data were compared with Student's *t*-test when the data followed a Gaussian distribution; nonnormally distributed data were compared with the Mann–Whitney *U* test when it did not follow Gaussian distribution. The Wilcoxon signed-rank test was used for the nonparametric paired data of two-group analysis. Multiple groups were compared with two-way ANOVA or one-way ANOVA followed by Tukey's multiple comparison test. For the data that did not follow a Gaussian distribution, we used robust two-way ANOVA by using R package WRS2. Statistical significance was set at *p* < 0.05.

## 3. Results

### 3.1. Nr2f2 Overexpression Aggravated Diabetic-Induced Heart Failure in Mice

**Figure 1:**
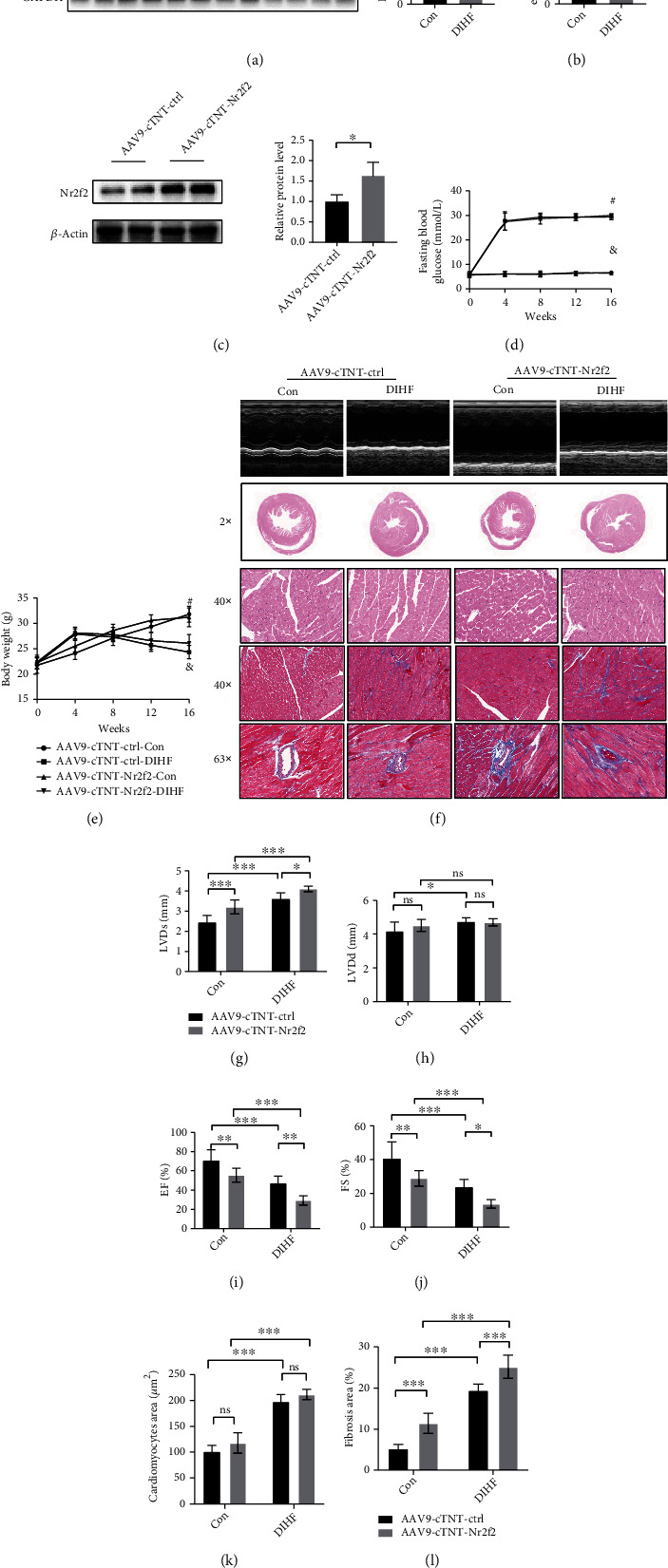
Overexpression of Nr2f2 aggravated the heart failure in diabetic mice. (a) Nr2f2 protein level in DCM mice was quantified by Western blot (*n* = 6, means ± SDs, ^∗∗^*p* < 0.001, Student' *t*-test). (b) Nr2f2 mRNA level in DCM mice were assessed by real-time qPCR (*n* = 6, means ± SDs, ^∗^*p* < 0.05, Student' *t*-test). (c) The overexpression efficiency of Nr2f2 was quantified by Western blot (*n* = 4, means ± SDs, ^∗^*p* < 0.05, Student' *t*-test). (d) Fasting blood glucose level of mice at each point (AAV9-cTNT-ctrl-Con, *n* = 10 vs. AAV9-cTNT-Nr2f2-Con, *n* = 10, ^&^*p* > 0.05; AAV9-cTNT-ctrl-DIHF, *n* = 8 vs. AAV9-cTNT-Nr2f2-DIHF, *n* = 6, ^#^*p* > 0.05). (e) Body weight of mice at each point (AAV9-cTNT-ctrl-Con, *n* = 10 vs. AAV9-cTNT-Nr2f2-Con, *n* = 10, ^#^*p* > 0.05; AAV9-cTNT-ctrl-DIHF, *n* = 8 vs. AAV9-cTNT-Nr2f2-DIHF, *n* = 6, ^&^*p* > 0.05). (f) Typical echocardiography, HE staining (20x and 400x magnification) and Masson staining (400x and 630x magnification) obtained from mice. (g–j) Echocardiography analysis of EF%, FS%, LVDd (mm), and LVDs (mm). (k) Cardiomyocytes area (*μ*m^2^). (l) Fibrosis area (%) (AAV9-cTNT-ctrl-Con *n* = 10, AAV9-cTNT-ctrl-DIHF *n* = 8, AAV9-cTNT-Nr2f2-Con *n* = 10, and AAV9-cTNT-Nr2f2-DIHF *n* = 6; means ± SDs, two-way ANOVA, ^∗^*p* < 0.05, ^∗∗^*p* < 0.01, and ^∗∗∗^*p* < 0.005, two-way ANOVA).

Protein levels of Nr2f2 were significantly 6-fold, and the mRNA level was approximately 2.5-fold increased in heart tissue of DIHF mice compared to Con mice (Figures 1(a) and 1(b)). Nr2f2 overexpression (Figure 1(c)) had no effect on fasting blood glucose and body weight in DIHF or Con mice (Figures 1(d) and 1(e)). Cardiac function, reflected by decreased EF and FS and increased LVDs and LVDd in heart tissue (Figures 1(f)–1(i)), was significantly worse in AAV9-cTNT-Nr2f2-Con compared to AAV9-cTNT-ctrl-Con mice (EF%: 70.76 ± 11.20 vs. 55.46 ± 7.04; FS%: 40.72 ± 9.744 vs. 28.91 ± 4.592; LVDs: 2.447 ± 0.3412 vs. 3.208 ± 0.3517 mm; and LVDd: 4.175 ± 0.536 vs. 4.509 ± 0.356 mm), AAV9-cTNT-Nr2f2-DIHF compared to AAV9-cTNT-ctrl-DIHF mice (EF%: 47.09 ± 7.540 vs. 29.35 ± 4.800; FS%: 23.73 ± 4.612 vs. 13.78 ± 2.533; LVDs: 3.614 ± 0.2975 vs. 4.107 ± 0.1367 mm; and LVDd: 4.737 ± 0.223 vs. 4.696 ± 0.216 mm).

In an aspect of structural staining, DIHF mice presented obvious cardiac hypertrophy and interstitial and perivascular fibrosis compared with Con mice (fibrosis area: 11.42 ± 2.458 vs. 5.170 ± 1.082%). Moreover, Nr2f2 overexpression had no impact on cardiac hypertrophy but exacerbated fibrosis in DIHF mice and the fibrosis area of the AAV9-cTNT-Nr2f2-Con group was larger than that of AAV9-cTNT-ctrl-Con group (fibrosis area: 25.17 ± 2.843 vs. 19.37 ± 1.590) (Figures 1(f), 1(k), and 1(l)). Nr2f2 overexpression impairs the cardiac function and aggravates the left ventricular remodeling and cardiac dysfunction of diabetic mice.

### 3.2. Nr2f2 Overexpression Exacerbated Ferroptosis and Oxidative Stress in Diabetic Mice

**Figure 2:**
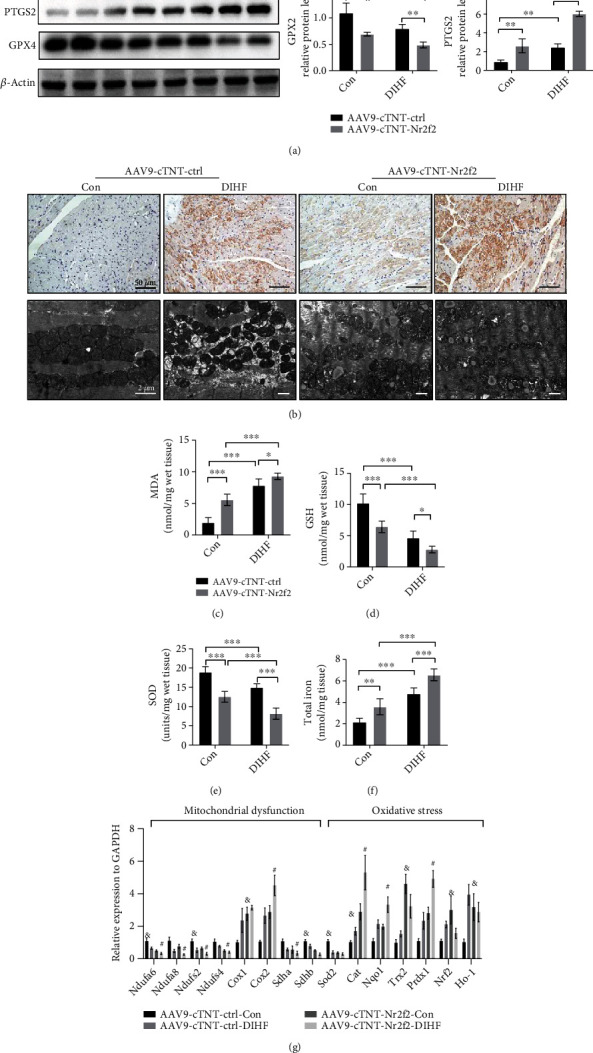
Overexpression of Nr2f2 aggravated cardiac ferroptosis. (a) Representative Western blot of PTGS2 and GPX4 (*n* = 4, means ± SDs, ^∗^*p* < 0.05 and ^∗∗∗^*p* < 0.005, ns: not significant, two-way ANOVA). (b) 4-HNE staining (scale bar = 50 *μ*m) and mitochondrial transmission electron microscopy (scale bar = 2 *μ*m) were identified in the heart tissue. (c–e) Tissue MDA, GSH, and SOD levels in each group (*n* = 6, means ± SDs, ^∗^*p* < 0.05 and ^∗∗∗^*p* < 0.005, two-way ANOVA). (f) Total iron level in each group (*n* = 6, means ± SDs, ^∗∗^*p* < 0.01 and ^∗∗∗^*p* < 0.005, two-way ANOVA). (g) Relative gene expression of mitochondrial function and oxidative stress (*n* = 6, means ± SDs, ^∗∗^*p* < 0.01 and ^∗∗∗^*p* < 0.005; ns: not significant, two-way ANOVA).

The previous study had demonstrated the activation of ferroptosis in diabetic cardiomyopathy [12]. In this study, we also found the upregulation of PTGS2 (approximately 2-fold) and downregulation of GPX4 (approximately half) in the AAV9-cTNT-ctrl-DIHF group compared with the AAV9-cTNT-ctrl-Con group (Figure 2(a)), as long as the MDA content was increased (1.953 ± 0.794 vs. 7.849 ± 1.027 nmol/mg wet tissue) and 4-HNE protein level decreased GSH the content (10.200 ± 1.476 vs. 4.633 ± 1.104 nmol/mg wet tissue) and SOD activity (18.930 ± 1.453 vs. 14.950 ± 0.982 units/mg wet tissue) (Figures 2(b)–2(f)). The characteristic morphological changes of mitochondria were shown by TEM, which mainly manifested as smaller mitochondria, a higher incidence of rupture of the outer mitochondrial membrane (Figure 2(b)). Seriously, compared with the AAV9-cTNT-ctrl-Con group, the AAV9-cTNT-Nr2f2-Con group exhibited strong evidence of ferroptosis, which is exacerbated in the AAV9-cTNT-Nr2f2-DIHF group. Additionally, Nr2f2 overexpression enhanced mitochondrial electron transport chain impairment and oxidative stress response, proved by decreased mRNA expression of *Ndufa6*, *Ndufa8*, *Ndufs2*, *Ndufs4*, *Sdha*, *Sdhb*, and *Sod2* and increased mRNA expression of *Cox1*, *Cox2*, *Cat*, *Nqo1*, *Trx2*, *Prdx1*, *Nrf2*, and *Ho-1* (Figure 2(g)).

### 3.3. Nr2f2-Knockdown Relieved PA-Induced Ferroptosis and Mitochondrial Dysfunction in NRCMs

**Figure 3:**
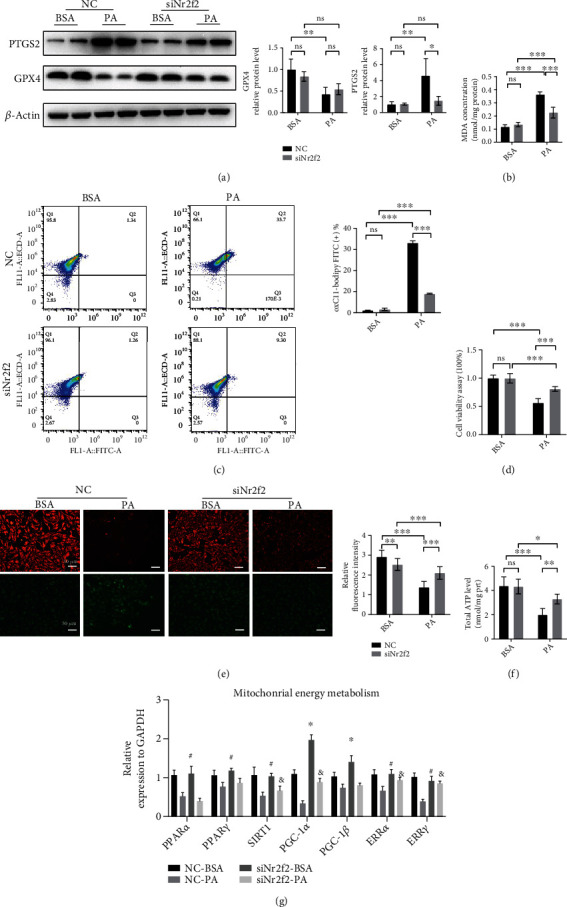
Knockdown of Nr2f2 alleviated ferroptosis induced by palmitic acid in neonatal rat cardiomyocytes. (a) Representative Western blot of PTGS2 and GPX4 (*n* = 4, means ± SDs, ^∗^*p* < 0.05 and ^∗∗^*p* < 0.01; ns: not significant, two-way ANOVA). (b) MDA level in each group (*n* = 6, means ± SDs, ^∗∗∗^*p* < 0.005; ns: not significant, two-way ANOVA). (c) Lipid ROS was assessed by C11 BODIPY 581/591 staining coupled with flow cytometry analysis (*n* = 3, means ± SDs, ^∗^*p* < 0.05 and ^∗∗∗^*p* < 0.005; ns: not significant, two-way ANOVA). (d) Cell viability was measured by CCK8 (*n* = 6, means ± SDs, ^∗∗∗^*p* < 0.005, two-way ANOVA). (e) Mitochondrial membrane potential was measured by JC-1 staining; red fluorescence represents healthy mitochondria, while green indicates loss of mitochondrial potential (scale bar = 50 *μ*m, *n* = 6, means ± SDs, ^∗∗∗^*p* < 0.005, two-way ANOVA). (f) Intracellular ATP level in each group (*n* = 6, means ± SDs, ^∗^*p* < 0.05, ^∗∗^*p* < 0.01, and ^∗∗∗^*p* < 0.005; ns: not significant, two-way ANOVA). (g) Relative expression of mitochondrial energy metabolic genes (*n* = 6, means ± SDs, NC-BSA vs. siNr2f2-BSA, ^#^*p* > 0.05 and ^∗^*p* < 0.05; NC-PA vs. siNr2f2-PA, ^&^*p* < 0.05, two-way ANOVA).

*In vitro*, PA treatment drove ferroptosis in NRCMs, which was reflected in approximately 4-fold increased PTGS2 expression, MDA level (0.118 ± 0.017 vs. 0.364 ± 0.020 nmol/mg protein), lipid-ROS level (1.143 ± 0.176 vs. 33.17 ± 0.924%), and decreased cell viability (1.000 ± 0.053 vs. 0.564 ± 0.072) compared with BSA treatment (Figures 3(a)–3(d)). Otherwise, Nr2f2 knockdown (Figure S1) partially alleviated PA-induced injury in NRCMs, by improving cell viability (0.998 ± 0.083 vs. 0.807 ± 0.043), influencing PTGS2 and GPX4 expression and decreasing MDA (0.135 ± 0.017 vs. 0.226 ± 0.041 nmol/mg protein) and lipid-ROS level (1.677 ± 0.520 vs. 9.047 ± 0.230%) compared to negative control (Figures 3(a)–3(d)).

Given the effect of Nr2f2 on mitochondrial function, mitochondrial membrane potentials (MMPs) were monitored by JC-1 staining. PA treatment contributed to the decline in MMPs (2.921 ± 0.337 vs. 1.413 ± 0.308) and the total cellular ATP level (4.368 ± 0.755 vs. 1.987 ± 0.514 nmol/mg protein) but Nr2f2 knockdown alleviated the mitochondrial dysfunction induced by PA (Figures 3(e) and 3(f)). Thus, we examined the genes of mitochondrial energy metabolism and found that PA treatment caused decreased expression of *PPARα*, *PPARγ*, *SIRT1*, *PGC-1α*, *PGC-1β*, *ERRα*, and *ERRγ* and Nr2f2 knockdown promoted the mRNA expression of *PGC-1α* and *PGC-1β* (Figure 3(g)).

### 3.4. PGC-1*α* Acts Downstream of Nr2f2 in Regulating Ferroptosis and Mitochondrial Dysfunction

**Figure 4:**
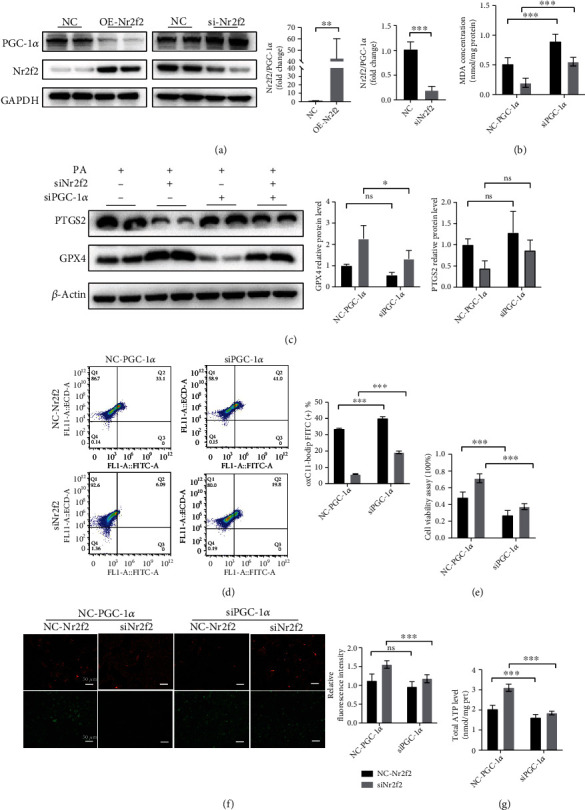
Nr2f2 regulates ferroptosis and mitochondrial dysfunction via PGC-1*α*. (a) Representative Western blot of the PGC-1*α* protein level after Nr2f2 overexpression and knockdown (*n* = 4, means ± SDs, ^∗∗^*p* < 0.01 and ^∗∗∗^*p* < 0.005, Student' *t*-test). (b) MDA level in each group (*n* = 6, means ± SDs, ^∗∗∗^*p* < 0.005; ns: not significant, two-way ANOVA). (c) Representative Western blot of PTGS2 and GPX4. (*n* = 4, means ± SDs, ^∗^*p* < 0.05 and ^∗∗^*p* < 0.01, two-way ANOVA). (d) Lipid ROS was assessed by C11 BODIPY 581/591 staining coupled with flow cytometry analysis (*n* = 3, means ± SDs, ^∗∗∗^*p* < 0.005, two-way ANOVA). (e) Cell viability was measured by CCK8 (*n* = 6, means ± SDs, ^∗∗∗^*p* < 0.005, two-way ANOVA). (f) Representative JC-1 staining (scale bar = 50 *μ*m, *n* = 6, means ± SDs; ns: not significant, ^∗∗∗^*p* < 0.005, two-way ANOVA). (g) Intracellular ATP level in each group (*n* = 6, means ± SDs, ^∗∗∗^*p* < 0.005, two-way ANOVA).

PGC-1*α* is an important coregulator of mitochondrial energy metabolism and oxidative stress. In order to investigate the regulatory relationship between Nr2f2 and PGC-1*α*, we constructed the Nr2f2 overexpression plasmid and found that Nr2f2 overexpression (Figure S2) negatively regulated PGC-1*α* in NRCMs (Figure 4(a)). Further, PGC-1*α* knockdown (Figure S3) exacerbated the ferroptosis and mitochondrial dysfunction induced by PA treatment and partially diminished the protective effect of Nr2f2 downregulation in PA-treated NRCMs (Figures 4(b)–4(g)). Overall, Nr2f2, acting as an upstream factor, negatively regulates PGC-1*α* and promotes ferroptosis and mitochondrial dysfunction induced by PA treatment in NRCMs.

## 4. Discussion

Diabetes is associated with a high incidence of heart failure and cardiac death, resulting in substantial morbidity and mortality in individuals [20]. Currently, there are no effective treatments for diabetes-induced heart failure (DIHF). Except for antidiabetes, the management of heart failure is also important. In the present study, we investigated the role of Nr2f2 in regulating ferroptosis in diabetes-induced heart failure mice by feeding a high-fat diet and streptozotocin intraperitoneal injection and the underlying mechanism. Findings revealed that Nr2f2 is increased in DIHF mice and Nr2f2 overexpression aggravated heart failure and promoted ferroptosis and mitochondrial dysfunction. Nr2f2 knockdown attenuated the ferroptosis and mitochondrial dysfunction induced by PA in NRCMs.

As a regulator of transcription, Nr2f2 plays an important role in many pathological and physiological processes, including organ development, metabolism, and cancer. Previous studies demonstrated that Nr2f2 is necessary for the development and functioning of the cardiovascular system. Altered Nr2f2 expression may lead to congenital heart defects (CHD) and susceptibility to atherosclerosis due to the impact of Nr2f2 on lipid metabolism and inflammation [16, 21, 22]. Specifically, Nr2f2 overexpression appears detrimental to the cardiovascular system by a mechanism linked to oxidative stress and mitochondrial dysfunction [14, 17]. Mice overexpressing Nr2f2 eventually develop heart failure due to impaired glycolipid metabolism and mitochondrial dysfunction [14]. In this study, we revealed that Nr2f2 expression was increased in heart tissue of DIHF mice and mice overexpressing Nr2f2 exhibited symptoms of severe heart failure. Recent evidence suggests that inhibition of Nr2f2 with a small molecular compound could suppress the progression and metastasis of prostate cancer [23], which reflects the application prospect of targeting Nr2f2.

Suppression of programmed cell death may be an effective strategy in the treatment of cardiovascular disease [24]. Distinct types of cell death that lead to cardiac contractile dysfunction and remodeling have been implicated in diabetes-induced heart failure. Long-term hyperglycemia can lead to apoptosis of renal cells and cardiomyocytes in patients with diabetes [25]. Ferroptosis, an iron-dependent, nonapoptotic kind of cell death, is characterized by lipid ROS accumulation and mitochondrial iron overload [26]. Accumulating evidence shows the contribution of ferroptosis to the development of cardiovascular diseases, and inhibition of ferroptosis may relieve diabetic myocardial ischemic reperfusion injury [7, 27, 28] and cardiomyopathy [12]. NRF2 (nuclear factor-erythroid 2 p45-related factor 2), another crucial transcription factor, is essential for preventing oxidative damage in diabetic cardiomyopathy by modulating several genes involved in ferroptosis, including those required for iron metabolism and lipid peroxidation [29]. Although, it is very close in terms of nomenclature, no direct evidence is available to indicate that the elevated Nr2f2 and cardiac ferroptosis are related. In our present study, diabetic mice overexpressing Nr2f2 showed obvious ferroptosis and an enhanced oxidative stress response in heart tissue, including upregulated *NRF2/HO-1* gene expression, which is involved in the intracellular iron metabolism and ferroptosis in doxorubicin-induced cardiomyopathy [8, 30, 31].

Mitochondria play a critical role in metabolism and energy regulation. As a major site of iron metabolism and ROS production, mitochondria likely contribute to ferroptosis [18, 32]. Ferroptosis is characterized by changes in mitochondrial morphology, with smaller than normal mitochondria and decreased mitochondria cristae. Diabetes-induced heart failure is associated with altered fatty acid metabolism and mitochondrial oxidative stress, such that targeting mitochondria has become a popular therapeutic strategy [33, 34]. Evidence suggests that Nr2f2 overexpression disrupts mitochondrial pathways [14]. In the present study, we verified that Nr2f2 knockdown could relieve mitochondrial damage and ferroptosis in PA-treated NRCMs. This study concludes that Nr2f2 may promote ferroptosis by affecting intracellular mitochondrial function, although the role of mitochondria in ferroptosis has been a matter of considerable debate. However, further research is required to confirm the association between PA-induced ferroptosis and mitochondrial dysfunction in NRCMs.

Previous reports showed that Nr2f2 directly modulates genes in the mitochondrial metabolic regulators, including ERR*α*, ERR*γ*, PPAR*α*, and PGC-1*α* [14]. In this study, we found that Nr2f2 knockdown mitigated PA-induced mitochondrial dysfunction by activating PGC-1*α* and PGC-1*β*. PGC-1*α* is a coregulator of mitochondrial metabolism, specifically involved in lipid and fatty acid metabolism. In the cardiovascular system, PGC-1*α* may regulate cardiomyocyte metabolism by coactivating PPARs, ERRs, and NRFs [35]. In diabetes, PGC-1*α* expression is downregulated resulting in cardiac complications [36]. PGC-1*α* knockout in mouse heart may damage the mitochondrial ETC and decrease ATP synthesis [37]. In this single study, Nr2f2 overexpression was associated with reduced PGC-1*α* expression, while suppressing Nr2f2 was associated with PGC-1*α* upregulation, suggesting that PGC-1*α* may serve as a crucial downstream target of Nr2f2. One of this study's limitations is that we did not investigate the function of PGC-1*β* in regulating ferroptosis which needed to further study.

## 5. Conclusions

In summary, our data indicate that expression of Nr2f2 was elevated in diabetic failing heart and Nr2f2 overexpression accentuated symptoms of heart failure and ferroptosis in diabetic mice. Nr2f2 knockdown mitigated the ferroptosis and mitochondrial dysfunction induced by PA treatment of NRCMs by downstream regulatory actions on PGC-1*α*.

## Figures and Tables

**Table 1:** Primer sequence.

Primer name	Sequence (5′-3′)
Mus-GAPDH-forward	AGGTCGGTGTGAACGGATTTG
Mus-GAPDH-reverse	TGTAGACCATGTAGTTGAGGTCA
Mus-Nr2f2-forward	TCAACTGCCACTCGTACCTG
Mus-Nr2f2-reverse	CCATGATGTTGTTAGGCTGCAT
Mus-Ndufa6-forward	TCGGTGAAGCCCATTTTCAGT
Mus-Ndufa6-reverse	CTCGGACTTTATCCCGTCCTT
Mus-Ndufa8-forward	GGAGCTGCCAACTCTGGAAG
Mus-Ndufa8-reverse	CCAGCGGCACAGCATAAAC
Mus-Ndufs2-forward	CAGCCAGATATTGAATGGGCA
Mus-Ndufs2-reverse	TGTTGGTCACCGCTTTTTCCT
Mus-Ndufs4-forward	CTGCCGTTTCCGTCTGTAGAG
Mus-Ndufs4-reverse	TGTTATTGCGAGCAGGAACAAA
Mus-COX2-forward	TTCAACACACTCTATCACTGGC
Mus-COX2-reverse	AGAAGCGTTTGCGGTACTCAT
Mus-COX1-forward	ATGAGTCGAAGGAGTCTCTCG
Mus-COX1-reverse	GCACGGATAGTAACAACAGGGA
Mus-Sdha-forward	GGAACACTCCAAAAACAGACCT
Mus-Sdha-reverse	CCACCACTGGGTATTGAGTAGAA
Mus-Sdhb-forward	AATTTGCCATTTACCGATGGGA
Mus-Sdhb-reverse	AGCATCCAACACCATAGGTCC
Mus-SOD2-forward	CAGACCTGCCTTACGACTATGG
Mus-SOD2-reverse	CTCGGTGGCGTTGAGATTGTT
Mus-CAT-forward	AGCGACCAGATGAAGCAGTG
Mus- CAT-reverse	TCCGCTCTCTGTCAAAGTGT
Mus-Nqo1-forward	AGGATGGGAGGTACTCGAATC
Mus-Nqo1-reverse	AGGCGTCCTTCCTTATATGCT
Mus-Trx2-forward	TGGGCTTCCCTCACCTCTAAG
Mus-Trx2-reverse	CCTGGACGTTAAAGGTCGTCA
Mus-Prdx1-forward	AATGCAAAAATTGGGTATCCTGC
Mus-Prdx1-reverse	CGTGGGACACACAAAAGTAAAGT
Mus-Nrf2-forward	TCTTGGAGTAAGTCGAGAAGTGT
Mus-Nrf2-reverse	GTTGAAACTGAGCGAAAAAGGC
Mus-HO-1-forward	AAGCCGAGAATGCTGAGTTCA
Mus- HO-1-reverse	GCCGTGTAGATATGGTACAAGGA
RAT-GAPDH-forward	GACTCTCCCACGGCAAGTT
RAT-GAPDH-reverse	GGTGATGGGTTTCCCGTTGA
RAT-PPAR*α*-forward	TGCGACATCATGGAACCCAA
RAT-PPAR*α*-reverse	CACAATCCCCTCCTGCAACT
RAT-PPAR*γ*-forward	GCCCACCAACTTCGGAATCA
RAT-PPAR*γ*-reverse	GCTGGAGAAATCAACCGTGG
RAT-SIRT1-forward	AGTTGCCACCAACACCTCTT
RAT-SIRT1-reverse	CCGGTCTGTCAGCATCATCTT
RAT-PGC-1*α*-forward	TTCGGTCATCCCAGTCAAGC
RAT-PGC-1*α*-reverse	CTGAAGTTGCCATCCCGTAGT
RAT-PGC-1*β*-forward	CAGAGGGCCCTGTTCAGATG
RAT-PGC-1*β*-reverse	ATACCACACGACCTTCACCG
RAT-ERR*α*-forward	TGGGCATCGAGCCTCTCTAC
RAT-ERR*α*-reverse	ACCGGGGGTTCAGTCTCAG
RAT-ERR*γ*-forward	TGGAGGCTGTCCAGAAACTT
RAT-ERR*γ*-reverse	TTGTGCATGGGCACTTTGC

## Data Availability

All data and figures used to support the findings of this study are included within the article.

## Abbreviations

DIHF: Diabetes-induced heart failure
Nr2f2: Nuclear receptor subfamily 2, group F, member 2
PGC-1*α*: Peroxisome proliferator-activated receptor-*γ* coactivator-1*α*
HF: Heart failure
T1DM: Type 1 diabetes
T2DM: Type 2 diabetes
DCM: Diabetic cardiomyopathy
COUP-TFII: Chicken ovalbumin upstream promoter-transcription factor II
ROS: Reactive oxygen species
TCA: Tricarboxylic acid cycle
PA: Palmitic acid
NRCMs: Neonatal rat cardiomyocytes
STZ: Streptozotocin
EF: Ejection fraction
FS: Fraction shortening
LVDs: Left ventricular systolic diameter
LVDd: Left ventricular diastolic diameter
GPX4: Glutathione peroxidase 4
PTGS2: Prostaglandin-endoperoxide synthase 2
4-HNE: 4-Hydroxynonenal
qRT-PCR: Real-time quantitative reverse transcription polymerase chain reaction
MDA: Malondialdehyde
GSH: Glutathione
SOD: Superoxide dismutase
AAV9: Adeno-associated virus 9
BSA: Bovine serum albumin
MMP: Mitochondrial membrane potential
MI: Myocardial infarction
IR/I: Ischemia reperfusion injury
Ndufa6: NADH:ubiquinone oxidoreductase subunit A6
Ndufa8: NADH:ubiquinone oxidoreductase subunit A8
Ndufs2: NADH:ubiquinone oxidoreductase core subunit S2
Ndufs4: NADH:ubiquinone oxidoreductase core subunit S4
Sdha: Succinate dehydrogenase complex flavoprotein subunit A
Sdhb: Succinate dehydrogenase complex flavoprotein subunit B
Sod2: Superoxide dismutase 2
Cox1: Cytochrome c oxidase subunit 1
Cox2: Cytochrome c oxidase subunit 2
Cat: Catalase
Nqo1: NAD(P)H quinone dehydrogenase 1
Trx2: Thioredoxin protein 2
Prdx1: Peroxiredoxin 1
Nrf2: Nfe2-like bZIP transcription factor 2
Ho-1: Heme oxygenase 1
PPAR*α*: Peroxisome proliferator-activated receptor alpha
PPAR*γ*: Peroxisome proliferator-activated receptor gamma
PGC-1*β*: Peroxisome proliferator-activated receptor-*γ* coactivator-1*β*
ERR*α*: Estrogen-related receptor alpha
ERR*γ*: Estrogen-related receptor gamma
SIRT1: Sirtuin 1.

## Supplementary Materials

Supplementary Materials S1 representative Western blot of Nr2f2 expression after Nr2f2 knockdown (*n* = 3, means ± SDs, ^∗∗^*p* < 0.01, Student' *t*-test). S2: relative mRNA expression of Nr2f2 expression after Nr2f2 overexpression (*n* = 4, means ± SDs, ^∗∗∗^*p* < 0.005, Student' *t*-test). S3: relative mRNA expression of PGC-1*α* after PGC-1*α* knockdown (*n* = 4, means ± SDs, ^∗∗∗^*p* < 0.005, Student' *t*-test).
