# Supplementary material for: Nr2f2 Overexpression Aggravates Ferroptosis and Mitochondrial Dysfunction by Regulating the PGC-1α Signaling in Diabetes-Induced Heart Failure Mice
Source: Mediators Inflamm. 2022 Aug 30;2022:8373389. doi: 10.1155/2022/8373389 (PMC9448590; doi:10.1155/2022/8373389)
Supplement: Supplementary Materials — S1 representative Western blot of Nr2f2 expression after Nr2f2 knockdown (n = 3, means ± SDs, ∗∗p < 0.01, Student' t-test). S2: relative mRNA expression of Nr2f2 expression after Nr2f2 overexpression (n = 4, means ± SDs, ∗∗∗p < 0.005, Student' t-test). S3: relative mRNA expression of PGC-1α after PGC-1α knockdown (n = 4, means ± SDs, ∗∗∗p < 0.005, Student' t-test). [file 8373389.f1.docx]

Supplementary figure

S1. Representative western blot of Nr2f2 expression after Nr2f2 knockdown. (n=3, means± SDs, ***p*<0.01, Student’ t test). S2. Relative mRNA expression of Nr2f2 expression after Nr2f2 overexpression. (n=4, means± SDs, ****p*<0.005, Student’ t test). S3. Relative mRNA expression of PGC-1α after PGC-1α knockdown. (n=4, means± SDs, ****p*<0.005, Student’ t test).
